# NSAIDs affect dendritic cell cytokine production

**DOI:** 10.1371/journal.pone.0275906

**Published:** 2022-10-13

**Authors:** Tonke K. Raaijmakers, Renske J. E. van den Bijgaart, Gert Jan Scheffer, Marleen Ansems, Gosse J. Adema

**Affiliations:** 1 Department of Radiation Oncology, Radiotherapy & OncoImmunology Laboratory, Radboud Institute for Molecular Life Sciences, Radboud UMC, Nijmegen, The Netherlands; 2 Department of Anesthesiology, Pain and Palliative Medicine, Radboud UMC, Nijmegen, The Netherlands; University of Bergen, NORWAY

## Abstract

**Background:**

Immunotherapy is now considered as the new pillar in treatment of cancer patients. Dendritic cells (DCs) play an essential role in stimulating anti-tumor immune responses, as they are capable of cross-presenting exogenous tumor antigens in MHCI complexes to activate naïve CD8+ T cells. Analgesics, like non-steroid anti-inflammatory drugs (NSAIDs), are frequently given to cancer patients to help relieve pain, however little is known about their impact on DC function.

**Methods:**

Here, we investigated the effect of the NSAIDs diclofenac, ibuprofen and celecoxib on the three key processes of DCs required for proper CD8+ cytotoxic T cell induction: antigen cross-presentation, co-stimulatory marker expression, and cytokine production.

**Results:**

Our results show that TLR-induced pro- and anti-inflammatory cytokine excretion by human monocyte derived and murine bone-marrow derived DCs is diminished after NSAID exposure.

**Conclusions:**

These results indicate that various NSAIDs can affect DC function and warrant further investigation into the impact of NSAIDs on DC priming of T cells and cancer immunotherapy efficacy.

## Introduction

Cancer patients often receive analgesics to treat acute or chronic cancer-related pain [1]. In many cases, non-opioids are sufficient to relieve pain, especially when the medication is taken regularly, and pain can be controlled and maintained [2]. Non-steroid anti-inflammatory drugs (NSAIDs) are a specific class of analgesics. NSAIDs are not recommended during chemotherapy as they can cover up a fever. Similarly, they are not advised closely before and after surgery because of their anti-coagulant effects [2]. However, their analgesic effect [3, 4], their effect on the immune system and on tumor progression deems them as interesting analgesic candidates for cancer patients. NSAIDs exert their function through inhibition of cyclooxygenase (COX) enzymes (COX1 and/or COX2). COX1 is a constitutively expressed enzyme, whereas COX2 is an inducible enzyme and increases upon cell activation during inflammatory processes, e.g. by pro-inflammatory cytokines, as well as damage-associated molecular patterns [5]. Non-selective NSAIDs inhibit the activity of both COX1 and COX2 (e.g. diclofenac and ibuprofen), while selective NSAIDs specifically target COX2 (celecoxib). COX enzymes convert arachidonic acid to prostanoids, including thromboxane and prostaglandins (PGE), in lipid droplets [6]. High COX expression levels in the tumor therefore often correlate with overproduction of PGE [7, 8]. The prostaglandin E2 (PGE2) lowers nociceptor thresholds [9], promotes tumor development and progression by evasion of apoptosis [10, 11], sustains proliferation [12, 13], induces angiogenesis [14–16], and promotes cancer cell adhesion, migration, and invasion [17]. Furthermore, PGE2 acts as a major mediator in inflammatory responses [18], mainly promoting immunosuppressive environments [19]. High COX levels in tumors [20–26] have been linked to lower survival rates [27, 28]. The use of NSAIDs in turn has been linked to lower cancer incidence [29–33], lower mortality [34, 35], and increased anti-tumor effects [22, 36, 37].

The immune system plays an essential role in the elimination of tumor cells. Initiation of the adaptive arm of the immune system, and specifically the activation of CD8+ cytotoxic T lymphocytes (CTL), is important, as these cells are able to recognize and kill tumor cells specifically. Dendritic cells (DCs) play a crucial role in orchestrating anti-tumor immunity as they are specialized cross-presenting cells, capable of priming tumor-specific CTLs. Three signals crucial for efficient T cell priming are: (cross)-presentation of extracellular tumor antigens in MHCI-molecules, co-stimulation, and cytokines [38–40]. Antigen presenting DCs are able to provide these signals, as they phagocytose tumor debris, cross-present these antigens and mature (express co-stimulatory markers) while migrating to the lymph nodes, and produce cytokines to activate naïve CD8+ T cells locally [41, 42]. Toll-like receptor (TLR) activation educates DCs to initiate effector T cell differentiation and expansion [43]. The immunomodulatory effect of several NSAIDs has been explored [44–47], but further insight is needed into the effect of different NSAIDs on the three signals exploited by DCs in one system.

Here, we set out to explore the effects of non-selective COX inhibitors diclofenac and ibuprofen and specific COX2 inhibitor celecoxib on the cross-presentation, co-stimulatory potential, and cytokine production by DCs. We report that TLR-induced cytokine production by murine bone-marrow derived DCs (mBMDCs) and human moDCs is impaired upon preincubation with NSAIDs. Understanding the role of NSAIDs on DC functioning might further improve DC-based cancer immunotherapy.

## Material and methods

### Mice, cell lines, and murine and human DC cultures

Female *C57BL/6J mice* (6–8 weeks old) were purchased from Charles River Wiga (Sulzfeld, Germany). Drinking water and standard laboratory food pellets were provided *ad libitum* and mice were allowed to settle for at least 1 week. The experiments were approved by the Animal Experiments Committee of the Radboud University Medical Center, and were performed in accordance with institutional, national and European guidelines. All mice were maintained under specific pathogen-free barrier conditions at the Central Animal Laboratory (Nijmegen, The Netherlands). Mice were sacrificed by cervical dislocation. mBMDCs were cultured as according to previously described protocol [48, 49]. In short, bone marrow was flushed out of tibia and femur and mashed over a nylon mesh with pore size 100 μm. Cells (4x10^6^ in 10 cm dish) were cultured for 8 days in RPMI-1640 (Gibco, #42401–018), supplemented with 10% heat-inactivated fetal bovine serum (FBS, Greiner Bio-One), 2mM L-glutamine (Lonza, #BE17-605E/U1), 1% penicillin/streptomycin (pen/strep, Gibco, #15140–122) and 50 μM beta-mercaptoethanol (Gibco, #21985–023) (BMDC medium) in the presence of 20 ng/ml rmGM-CSF (PeproTech, # 315–03), at 37°C with 5% CO_2_. On day 3 and 6, rmGM-CSF was added to the culture. Non-adherent cells were harvested and used for assays (mBMDCs). B3Z cells, a T cell hybridoma specific for the immunodominant OVA K^b^ peptide in H-2K^b^, which carries a β-galactosidase construct driven by NF-AT elements from the interleukin-2 (IL-2) promotor [50], were cultured in IMDM (Gibco, #21980–032) supplemented with 5% FBS, 2 mM L-glutamine, 1% pen/strep, 50 μM beta-mercaptoethanol, and 0.5 mg/ml hygromycin (Invitrogen, #10687010). Human monocyte-derived DCs (moDCs) were generated as described previously [51, 52], from cells isolated from buffy coats obtained from healthy volunteers (Sanquin, Nijmegen, The Netherlands) after written informed consent as per the Declaration of Helsinki. moDCs were acquired by culturing ficoll gradient (SepMate™, StemCell, #85450) obtained peripheral blood mononuclear cells (PBMCs) with 450 U/ml rhGM-CSF (Immunotools, #11343128) and 300 U/ml rhIL-4 (Immunotools, #11340047) for 6 days. rhGM-CSF and rhIL-4 were added again at day 3. moDCs were cultured in RPMI-1640 supplemented with 10% FCS, 2 mM glutamine, and 1% pen/strep. Adherent cells were used for assays. During all incubation steps, cells were kept at 37°C with 5% CO_2_, unless stated otherwise.

### Adjuvants, reagents and antibodies

Lipopolysaccharide (LPS) was purchased from Sigma-Aldrich (St Louis, MO, USA), and used at a concentration of 0.5 μg/ml. CpG-ODN 1668 (‘5-TCCATGACGTTCCTGATGCT-3’) with total phosphorothioate-modified backbone was purchased from Sigma Genosys (Haverhill, UK), and used at a concentration of 1 μg/ml. R848 was purchased from Enzo Life Sciences, and used at a concentration of 4 μg/ml. The NSAIDs diclofenac sodium salt (PHR1144-1G), celecoxib (PHR1683-1G), and ibuprofen (PHR-1004-1G) were purchased from Sigma-Adrich. For every experiment, a fresh dilution in medium was made. Celecoxib was first diluted in DMSO and thereafter further diluted in medium (DMSO concentration <0.03%). For flow cytometry experiments the following antibodies (clone name in brackets, followed by supplier, and dilution) conjugated to various fluorophores were used: MHCII-BV510 (M5/114.15.2, Antibodychain, 1:500), CD80-A488 (16-10A1, Antibodychain, 1:1000), CD11b-PerCP (M1/70, Biolegend, 1:600), CD115-PeCy7 (AFS98, eBioscience, 1:200), CD11c-APC (HL3, BD, 1:400), and CD86-APCCy7 (GL-1, Biolegend, 1:800). Viability dye used was eFluor™ 450 (ThermoFisher, 1:4000).

### RNA isolation and RT-qPCR

Total RNA was isolated 0.5x10^6^ mBMDCs stimulated with TLR for 6 or 18 hours (hr), using TRIzol reagent (Invitrogen Life Technologies) according to the manufacturer’s instructions, with minor modifications. mRNA levels for the genes of interest were determined with a CFX96 sequence detection system (Bio-Rad) using the Faststart SYBR green mastermix (Roche) with SYBR Green as the fluorophore and gene-specific oligonucleotide primers. Primers used are COX1 FW TTACTATCCGTGCCAGAACCA, COX1 REV CCCGTGCGAGTACAATCACA, COX2 FW TTCAACACACTCTATCACTGGC, COX2 REV AGAAGCGTTTGCGGTACTCAT, Rer1 FW GCCTTGGGAATTTACCACCT, Rer1 REV CTTCGAATGAAGGGACGAAA. Quantitative PCR data were analyzed with the CFX Manager V1.6.541.1028 software (Bio-Rad) and checked for correct amplification and dissociation of the products. mRNA levels of the genes of interest were normalized to mRNA levels of Rer1 and were calculated according to the cycle threshold method.

### In vitro cross-presentation assay

For in vitro cross-presentation assays 80x10^3^ NSAID preincubated mBMDCs were pulsed with 80 μg/ml of endotoxin-free chicken egg ovalbumin (OVA protein, Endograde, Hyglos GmbH, Germany) in the presence of 400 ng/ml immune stimulatory complexes (ISCOMs). After pulsing, cells were washed and cultured overnight with 80x10^3^ B3Z cells. As a control for cell viability and/or MHC-I expression levels, DCs were pulsed with 5 ng/ml OVA K^b^ peptide (SIINFEKL, 257–264, AS-60193, Tebu-bio) 30 min before adding the B3Z cells. The presentation of OVA K^b^ peptide in H-2K^b^ results in production of β-galactosidase (LacZ) by B3Z cells, which can be detected by adding 0.15 mM chlorophenolred-h-D-galactopyranoside (Calbiochem), 9 mM MgCl_2_, 0.125% NP40, and 7.5 mM DTT in PBS. Plates were incubated for 3 to 5 hr and absorbance values were measured at 595 nm using a photo spectrometer (Biorad).

### Flow cytometry

Expression of maturation markers was assessed using flow cytometry. 15x10^4^ NSAID preincubated mBMDCs were stimulated with TLR ligands, washed with medium, and rested overnight in fresh medium. Subsequently, medium was removed, and cells were washed with phosphate buffered saline (PBS). Samples were incubated with viability dye for 15 minutes on ice, and washed with PBS and thereafter washed with PBS supplemented with 0,5% bovine serum albumin and 0,05% sodium azide (PBA). Samples were incubated with Fc-block (CD16/32, clone 2.4G2, BD, 1:800) for 10 minutes on ice. Next, cells were incubated with fluorescently labeled antibodies in PBA for 20 min on ice. Subsequently, cells were washed twice, diluted in PBA and measured on FACS Canto.

### Measurement soluble factors

15x10^4^ NSAID preincubated mBMDCs were stimulated with TLR ligands, washed with medium, and rested overnight in fresh medium. Supernatant was collected 16–24 hr after incubation with adjuvants and stored at -80°C. Cytokines (IL-6, IL-10, IL-12, and TNF-α) were measured using ELISA kits (mIL-6: 88–7064, mIL-10: 88–7105, mIL-12p70: 88–7121, mTNF-α: 88–7324, hIL-6: 88–7066, hIL-10: 88–7106, hIL-12: 88–7126, hTNF-α: 88–7346; all from Invitrogen) according to the manufacturer’s instructions. PGE2 was measured using the R&D kit (KGE004B), according to manufacturer’s instructions, with minor modifications. In short, prior to the PGE2 measurement, large size proteins in the supernatant interfering with the assay were removed using a perchloric-acid (PCA) precipitation. For this, 13.7% PCA (SIGMA, 244252-100M) was added to supernatant. Supernatants were spun down for 5 minutes at maximum speed, aspirated to new plate, and neutralization was performed using 4N NaOH.

### Viability measurement

Metabolic activity as a measure for cell viability was assessed using the CCK8 kit (96992-3000TESTS-F, Sigma-Aldrich). In short, 15x10^4^ GMCSF DCs were cultured with NSAIDs for 6h. The cells were washed, medium was replaced (100 μL) and 20 μL CCK8 reagent was added to each well. Plates were incubated for 1 to 3 hr and absorbance values were measured at 450 nm using a photo spectrometer (Biorad).

### Statistical analysis

Depending on the experimental layout, data were analyzed using a two-tailed Student’s t-test, a one-way or 2-way ANOVA or mixed effects analysis with post hoc Dunnett’s or Sidak’s multiple comparisons test (medium versus rest) or Tukey’s multiple comparisons test, as indicated in the figure legends. Differences were considered significant when P values were smaller than 0.05, while the following symbols were used: *P < 0.05; **P < 0.01; ***P < 0.001. The statistical analyses were performed in Graphpad Prism 8.0.1.

## Results

**Fig 1:**
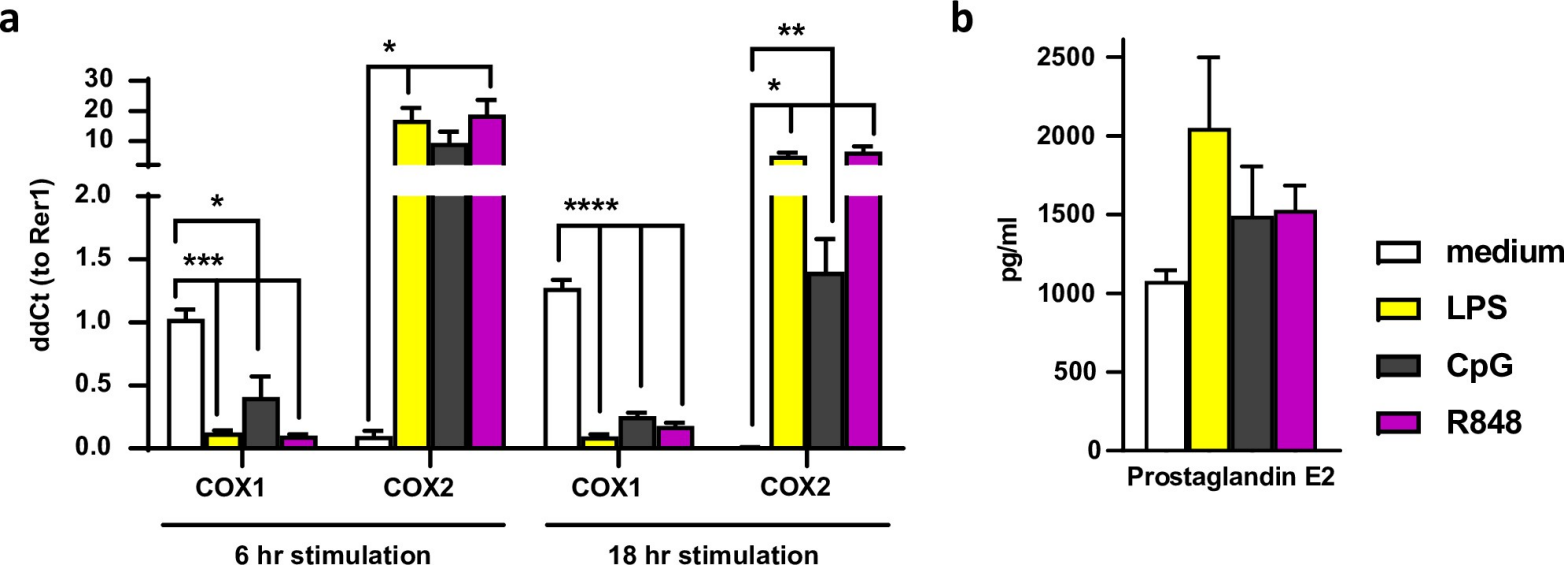
COX expression and PGE2 production upon TLR exposure. mBMDCs were treated for 6 hr (a) and 18 hr (a, b) with the TLR adjuvants LPS, CpG, and R848. (a) RT-QPCR was performed for mRNA expression of COX1 and COX2 (mBMDCs, n = 6). (b) PGE2 was measured using a competitive enzyme immunoassay in PCA-treated supernatant (n = 3). Results are shown as means with SEM. Statistical significance was calculated using a one-way ANOVA with Dunnett’s multiple comparison test, medium versus rest.

**Fig 2:**
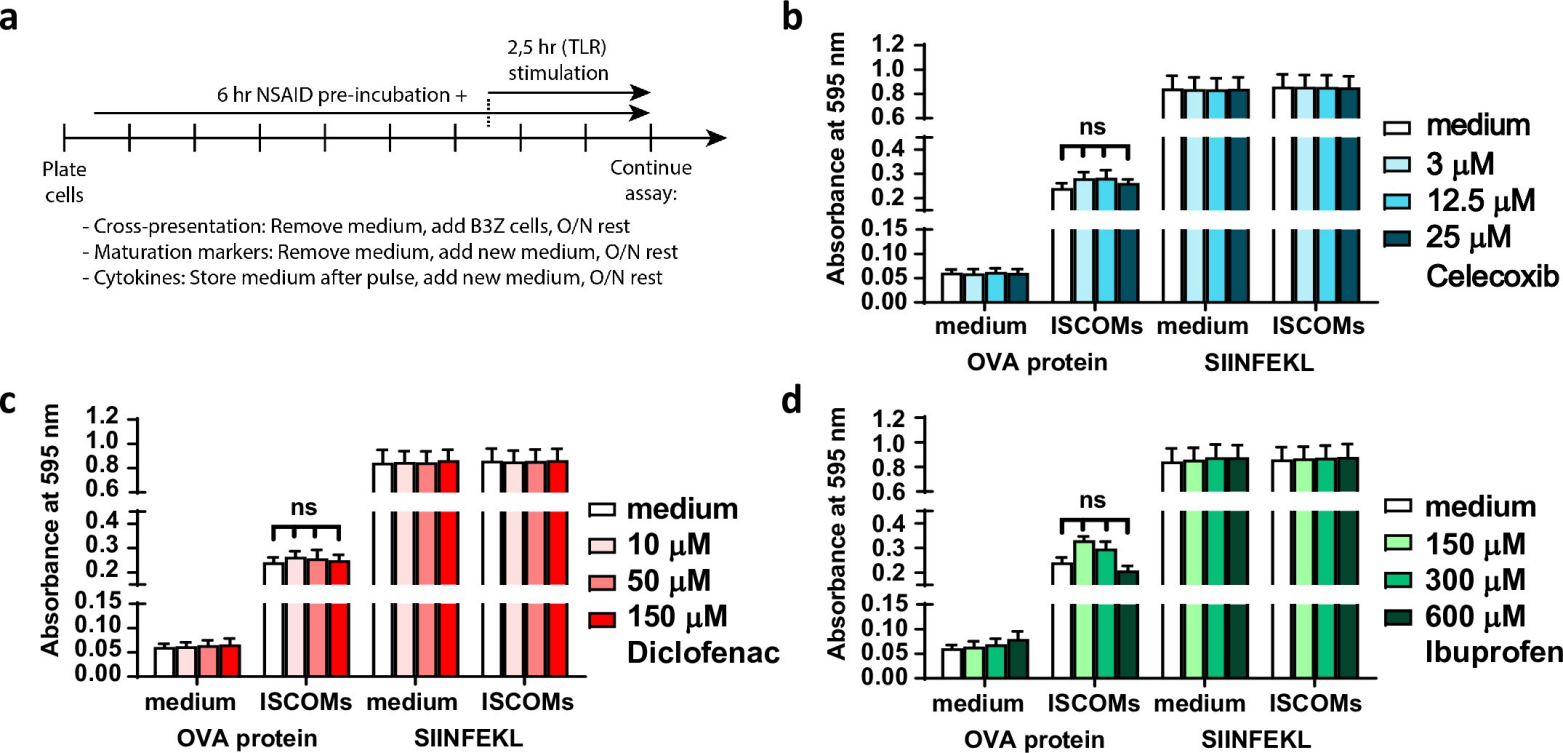
NSAIDs do not affect ISCOM induced cross-presentation. (a) Timeline shows the experimental setup for assessing cross-presentation, maturation markers and cytokine production by mBMDCs. (b-d) mBMDCs were first pretreated with NSAIDs for 6 hr, washed and subsequently treated with OVA protein and ISCOMs for 2.5 hr, and then co-cultured with B3Z T cells for 18 hr. As a positive control for viability and MHC-I levels, mBMDCs were pulsed with OVA peptide (SIINFEKL) 0.5 hr before coculture with B3Z T cells (n = 4). ISCOM induced cross-presentation of OVA protein and stable loading of exogenous SIINFEKL by celecoxib (b), diclofenac (c), and ibuprofen (d) pretreated DCs. Results are shown as means with SEM. Statistical significance was calculated using a one-way ANOVA with Dunnett’s multiple comparison test, medium versus rest.

TLR matured mBMDCs provide 3 key signals to induce CTLs: the presentation of extracellular (tumor) antigens in the MHCI complex, costimulation, and cytokines [38]. mBMDCs also respond to TLR triggering by a strong upregulation of COX2 mRNA expression, while COX-1 expression is down regulated (Fig 1A). This effect of TLR stimulation on the expression of COX enzymes in mBMDC is also reflected by the increased production of PGE2 (Fig 1B) and is in line with previous findings [50]. We then set out to explore the effect of NSAIDs on cross-presentation, costimulation and cytokine production by DCs. For this, we pretreated mBMDCs with NSAIDs for 6 hr, followed by a 2.5 hr induction of the DC cross-presentation adjuvant ISCOMs or a 2.5 hr induction of DC maturation by the TLR ligands LPS, CpG, or R848 (Fig 2A for experimental setup). This timing regimen was chosen as a 2.5 hr incubation with ISCOMs or TLR-ligands is sufficient for the induction of cross-presentation, co-stimulatory marker expression and cytokine secretion by DCs (S1 Fig). Exposure of DC for 6 hr to NSAIDs did not affect their viability (S2A Fig) nor the COX1 or COX2 expression (S2B Fig). A 6 hr pre-incubation period with NSAIDs allowed us to study the early effects of NSAIDs on DC function.

### NSAIDs do not affect ISCOM induced cross-presentation by mBMDCs

The effect of NSAIDs on the DC’s capacity to cross-present antigens was assessed using mBMDCs treated with the model antigen OVA in combination with the cross-presentation inducing ISCOM adjuvant as described previously [45]. Cross-presentation of the OVA peptide (SIINFEKL) in MHCI molecules by DCs was detected upon co-culture with the OVA-specific B3Z reporter T cell hybridoma as a readout system (Fig 2A). As expected, OVA protein cross-presentation by mBMDCs is greatly enhanced by the addition of ISCOMs (Fig 2B–2D). Preincubation of DCs with the NSAIDs celecoxib (Fig 2B), diclofenac (Fig 2C) or ibuprofen (Fig 2D) prior to exposure to OVA protein and ISCOMs did not affect cross-presentation of OVA protein. B3Z activation by OVA peptide loaded control DC was similar in all conditions, indicating that MHCI expression and viability was not affected by preincubation of the DCs with NSAIDs. These results demonstrate that NSAIDs do not affect ISCOM induced cross-presentation by mBMDCs.

### Maturation marker profiles remain unaffected by NSAID exposure

**Fig 3:**
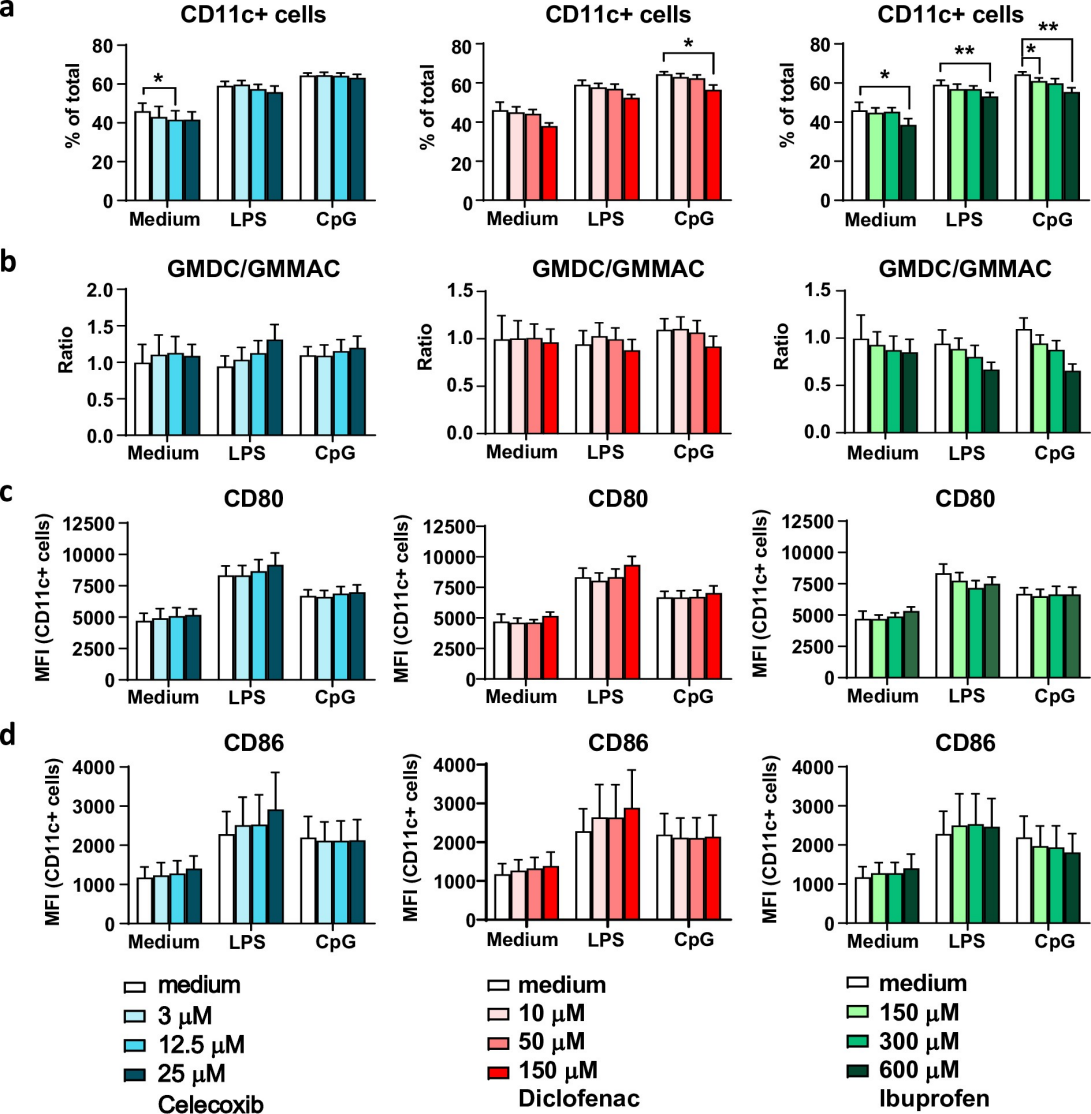
NSAIDs do not alter TLR-induced maturation marker expression. (a-d) mBMDCs were first pretreated with NSAIDs for 6 hr, followed by a 2.5 hr TLR stimulation with LPS, CpG, or R848. After overnight rest in fresh medium, composition of the mBMDC culture (a, b) and maturation marker expression (c, d) were analyzed using flow cytometry (n = 4). Results are shown as means with SEM. (a) Percentage CD11c+ cells in BMDC culture. (b) GM-DC/GM-MAC ratio within CD11c+ population. (c) Mean Fluorescent Intensity (MFI) ± SEM of CD80 and (d) CD86 in CD11c+ population. Statistical significance was calculated using a one-way ANOVA with Dunnett’s multiple comparison test, medium versus rest.

The bone-marrow-derived DC cultures with GM-CSF give rise to two major DC subpopulations [53], the GM-MAC (MHCII^low^CD11b^hi^CD115^hi^) and GM-DC (MHCII^hi^CD11b^int^CD115^low^). This prompted us to assess whether NSAIDs specifically affect the development or maturation of these different DC subpopulations (S3A Fig). Our flow cytometry data shows that the percentage of total CD11c+ cells was slightly lowered by the 6 hr diclofenac preincubation, when stimulated with CpG (Fig 3A). Preincubation with ibuprofen dose-dependently lowered CD11c+ population in all TLR-stimulated conditions (Fig 3A). The ratio between the subpopulations of GM-DCs versus GM-MACs was not significantly affected upon NSAID exposure (Fig 3B).

Next, we assessed the expression of maturation markers CD80 and CD86 on total CD11c+ DCs and the GM-DC subpopulation as this is the subpopulation expressing highest levels of CD80 and CD86 (S3B Fig) following TLR stimulation. CD80 and CD86 are important co-stimulatory molecules expressed on mature DCs that help the activation of CD8+ T cells towards effective CTLs. NSAIDs pretreatment did not significantly affect the expression levels of CD80 and CD86 on CD11c+ DCs (Fig 3C and 3D). In the GMDC subpopulation only minor differences were observed (S3C–S3E Fig). Together these results imply that preincubation with NSAIDs does not affect TLR-induced maturation marker expression.

### NSAID exposure reduces TLR-induced cytokine production by mBMDCs

**Fig 4:**
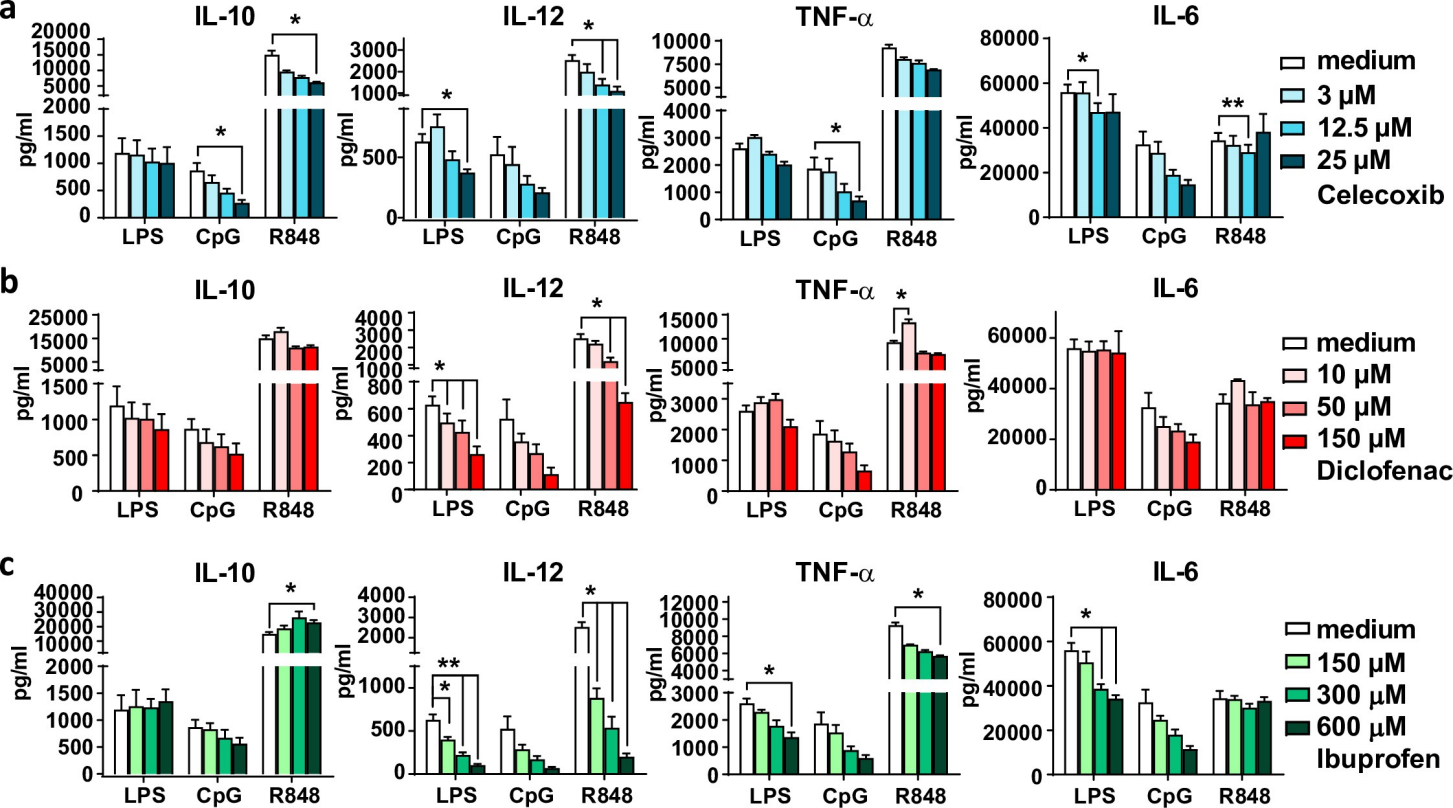
NSAID preincubation reduces TLR induced cytokine production. (a-c) mBMDCs were first pretreated with NSAIDs for 6 hr, followed by a 2.5 hr TLR stimulation with LPS, CpG, or R848. After overnight rest in fresh medium, cytokines were measured using ELISA (n = 4). IL-10, IL-12, TNF-α, and IL-6 production after TLR stimulation by (a) celecoxib, (b) diclofenac, and (c) ibuprofen preincubated DCs. Results are shown as means with SEM. Statistical significance was calculated using a one-way ANOVA with Dunnett’s multiple comparison test, medium versus rest.

Cytokine secretion by DCs is the third signal essential for T cell activation [54], and also plays an important role in the differentiation towards specific subtypes of T cells [55, 56]. Therefore, we investigated cytokine production by mBMDCs after preincubation with different NSAIDs and stimulation with different TLR adjuvants. As expected, DCs pulsed for 2.5 hr with adjuvants increase their overnight production of IL-6, IL-10, IL-12 and TNF-α (S4A Fig). Interestingly, preincubation with celecoxib reduced TLR-induced production of IL-10, IL-12, TNF-α, and IL-6 (Fig 4A). Diclofenac pre-exposure significantly decreased IL-12 production upon TLR stimulation (Fig 4B). Also a trend towards lower IL-10, IL-6 and TNF-α production after CpG stimulation by diclofenac pretreated DCs was observed. Ibuprofen also strongly inhibited IL-12 production of DCs (Fig 4C). In addition, also TNF-α and IL-6 production was decreased after ibuprofen preincubation (Fig 4C). These results indicate that preincubation with NSAIDs leads to decreased TLR-induced cytokine production by mBMDCs.

### Cytokine secretion by mature human DCs is affected by exposure to NSAIDs

**Fig 5:**
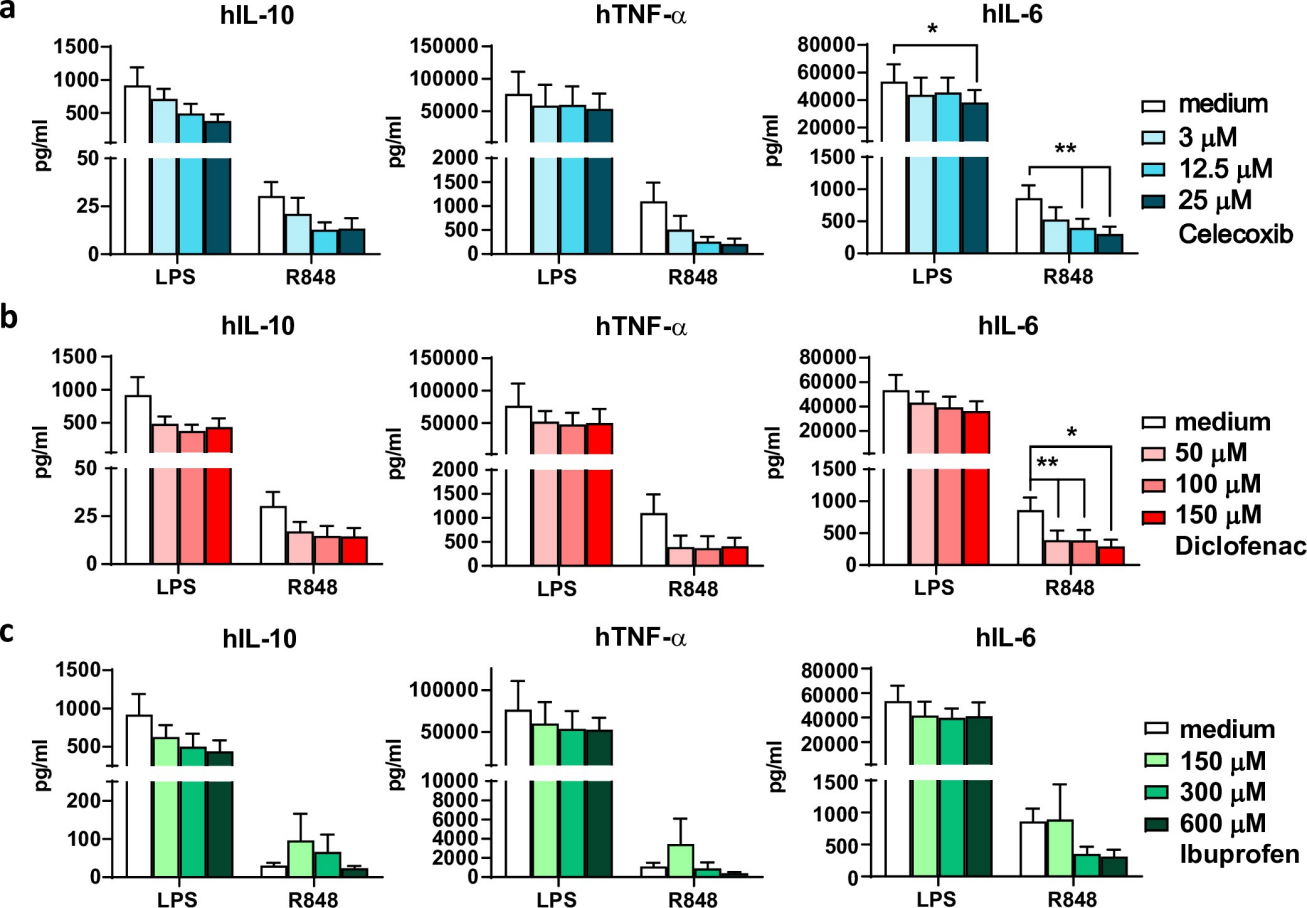
Decreased cytokine production by moDCs upon TLR stimulation when preincubated with NSAIDs. (a-c) moDCs were first pretreated with NSAIDs for 6 hr, followed by a 2.5 hr TLR stimulation with LPS or R848. After overnight rest in fresh medium, cytokines were measured using ELISA (n = 5–6). IL-10, TNF-α, and IL-6 production after TLR stimulation by (a) celecoxib, (b) diclofenac, and (c) ibuprofen preincubated moDCs. Results are shown as means with SEM. Statistical significance was calculated using Mixed-effects analysis with Dunnett’s multiple comparisons test.

To extrapolate our data on the effect of NSAIDs on cytokine production by mBMDCs to the human situation, human monocyte derived DCs (moDCs) were cultured from human blood and exposed to NSAIDs according to the same schedule (Fig 2A). Many reports have described that myeloid DCs and moDCs do not express TLR9 or do not respond to its ligand CpG [57–62]. We therefore used LPS and R848 as TLR stimuli. As expected, also human moDCs secrete IL-6, IL-10 and TNF-α after 2.5 hr stimulation with adjuvants (S4B Fig). TNF-α was mainly produced within the 2.5 hr stimulation period (S4B Fig) with R848, while IL-12 secretion was not significantly detected after LPS nor R848 stimulation (S4B Fig). In line with our murine data, also human moDCs show a trend towards diminished cytokine expression profile when exposed to celecoxib or diclofenac prior to the TLR stimulation (Fig 5A and 5B). The decreased production of TNF-α upon celecoxib or diclofenac preincubation was mainly detected in the first 2.5 hr during TLR stimulation (S4C Fig). For ibuprofen treated moDCs, the effect was less pronounced (Fig 5C). Altogether, TLR-stimulated human moDCs, like mBMDCs, appear to produce less cytokines when pretreated with celecoxib and diclofenac.

## Discussion

There is compelling evidence that NSAIDs lower different cancer types (colon, breast, prostate and lung) incidence and improve survival [29–35]. NSAIDs are suggested to play a role in the modulation of anti-tumor immunity [22, 36, 37, 63]. Since DCs play a vital role in the anti-tumor immunity cascade, we have studied the effects of NSAIDs on the three pathways (cross-presentation, co-stimulation, cytokines) applied by DCs to induce tumor-specific CD8+ CTLs. Our data demonstrate that celecoxib (selective COX2 inhibitor) and diclofenac and ibuprofen (COX1/2 inhibitors) have no or little effect on DC cross-presentation and CD80/CD86 co-stimulatory molecule expression, but reduce TLR-induced cytokine production by both murine bone marrow-derived DCs and human moDCs.

Cross-presentation is a process primarily executed by DCs and is essential to initiate a cytotoxic T cell response to exogenous tumor antigens. In this study, we show that diclofenac, ibuprofen and celecoxib had no effect on ISCOMs-induced cross-presentation and TLR-induced (murine) DC maturation. Interestingly, in contrast to our results on freshly differentiated DCs, Kim *et al*. has shown that immortalized DCs (DC2.4) are slightly restricted in cross-presentation of OVA present in biodegradable microspheres when cultured with ibuprofen [44]. Their basal CD80 and CD86 expression slightly increased upon 18 hr ibuprofen exposure but not their phagocytic capacity [44]. The use of a different source of DCs, antigen delivery vehicle and stimulation schedule (prolonged dosed and higher doses of ibuprofen) may explain these differences. In line with our data, diclofenac does not alter nickel-induced human DC maturation and CD86 expression [46].

Furthermore, we report that diclofenac strongly reduced the production of TLR-induced pro- and anti-inflammatory cytokines by murine DCs, and a similar trend towards reduced production was observed for human DCs. Statistical significance for NSAID-induced reduction in cytokine production by TLR stimulated moDCs was not reached, as the magnitude of the effects were variable between donors.

The secretion of distinct soluble and membrane-bound molecules determines the downstream polarization of T cells towards type 1 or type 2 responses [64]. Th1 cells secrete IL-2 and IFN-γ, cytokines promoting differentiation and maturation of CD8+ T cells into CTLs [65, 66]. Interfering with DC polarization and thereby cytokine production can thus affect T cell skewing and thereby modify the outcome of anti-tumor immune responses. Previous studies showed that diclofenac induces the polarization towards Th1 instead of Th2 cells, as it suppresses type 2 (CCL17) DC cytokine secretion [46], and not type 1 cytokines (TNF-α, IL-12p70). Another group showed that murine bone marrow-derived DCs co-cultured with glioma cells show enhanced IL-12 and decreased IL-10 secretion in the presence of diclofenac after stimulation with R848 [45]. Furthermore, *ex vivo* analyses revealed that tumor-infiltrating DCs regained their capacity to produce IL-12 on R848 stimulation after diclofenac exposure [45]. In contrast to our results where we saw a decrease in TLR-induced IL-12 production after pretreatment with diclofenac, these studies indicate that diclofenac promotes Th1 differentiation. This discrepancy possibly depends on the exposure time with NSAIDs, specific DC subsets used and the presence of glioma cells. The environmental context is important for the effect of NSAIDs on cytokine production by DCs. Future research will have to elucidate whether (tumor)microenvironmental factors influence pro- and anti-inflammatory cytokine production upon NSAID exposure.

Previously, it was shown that also celecoxib may modulate the balance between Th1 cytokines and Th2 cytokines by increasing Th1 cytokine IL-12 and reducing Th2 cytokine IL-10 [47], although its effect on cytokine production was most evidently seen in combination with tumor lysate. In contrast to the effect of celecoxib on tumor lysate induced DCs, we here show that celecoxib reduced TLR-induced production of IL-10, IL-12, TNF-α, and IL-6. It will be interesting to study the effect of altered cytokine production by NSAIDs through DCs on T cell skewing/differentiation in future experiments.

Our study underscores that combination of NSAIDs with DC-based immunotherapies should be explored in more detail, as NSAIDs are able to alter DC function, and thereby could affect (immuno)therapy outcome. Since NSAIDs also reduce immunosuppressive PGE2 levels in the tumor microenvironment, their combination with different immunotherapies has been studied preclinically. Both celecoxib and aspirin reduced PGE2 levels in the melanoma tumor microenvironment, inhibited PGE2-dependent suppression of myeloid cell activation, and increased anti-PD-1 efficacy [67]. Immature human DCs differentiate towards stable myeloid-derived suppressor cells (MDSC) when exposed to PGE2 [68]. Celecoxib refined DC-based immunotherapy by preventing the local and systemic expansion of all MDSC subtypes, reducing levels of reactive oxygen species and nitric oxide, and reversing T cell tolerance for mesothelioma [69]. Also in COX2 inhibitor SC5836-treated 4T1 bearing mice, MDSC accumulation in the tumor was reduced, and primary outgrowth was delayed [70]. Celecoxib administered after primary tumor establishment synergized with tumor lysate‐pulsed DCs and GM‐CSF [71]. This combination therapy suppressed primary 4T1 murine mammary tumor growth and markedly reduced the occurrence of lung metastases, due to an enhanced tumor-specific immune response [71].

The direct effect of NSAIDs on DC polarization may play a pivotal role in the initiation of anti-tumor immunity. Now that NSAIDs are explored for their beneficial properties in diverse anti-tumor immunotherapies in different tumor types, it will be important to further study their potential influence on treatment outcome.

## Conclusion

Altogether we show that preincubation with NSAIDs does not affect ISCOM-induced cross-presentation nor TLR-induced CD80 or CD86 co-stimulatory molecule expression. Cytokine production by both mBMDCs and human moDCs, however, is diminished when DCs were pretreated with celecoxib, diclofenac, or ibuprofen. Since the presence of NSAIDs can affect DC polarization, giving NSAIDs to cancer patients might affect the initiation of the anti-tumor immunity cascade, and thereby could affect treatment outcome.

## Supporting information

S1 Fig 2.5 hr stimulation induces cross-presentation, maturation markers, and sufficient cytokine production after replacement medium. (a) Experimental design with different incubation durations and stimuli. (b) mBMDCs were treated with OVA protein and ISCOMs for indicated durations, and then co-cultured with B3Z T cells for 18 hr. As a positive control for viability and MHC-I levels, mBMDCs were pulsed with OVA peptide (SIINFEKL) 0.5 hr before coculture with B3Z T cells (n = 3). Statistical significance calculated using 2-way ANOVA, Sidak’s multiple comparisons test (for medium versus ISCOM), Tukey’s multiple comparisons test (for ISCOMs versus ISCOMs). mBMDCs were stimulated with LPS, CpG, or R848 for indicated durations (c-e). After overnight rest in fresh medium (for 1, 2.5, and 5 hr pulse) or overnight stimulation without rest in fresh medium (O/N stimulation), maturation marker expression in CD11c+ cells was analyzed using flow cytometry (n = 5). Statistical significance was calculated using a one-way ANOVA with Dunnett’s multiple comparison test, medium versus rest. Directly after the pulse (d), and during the first and second 6 hr after refreshment of the medium, cytokines IL-6, IL-10, IL-12 and TNF-α were measured using ELISA (n = 2–3). (b-d) Results are shown as means with SEM.
(TIF)

S2 Fig Viability and COX1/2 expression is maintained after 6 hr NSAID stimulation. (a-b) mBMDCs were treated for 6 hr with NSAIDs. (a) CCK8 assay as a read out for cell viability and metabolic activity, after 6 hr stimulation with NSAIDs. Raw data–blanco depicted (n = 4). Statistical significance was calculated using one-way ANOVA with Dunnett’s multiple comparisons test, medium versus rest. (b) RT-QPCR was performed for mRNA expression of COX1 and COX2 (mBMDCs, n = 3–4). Results are shown as means with SEM. Statistical significance was calculated using a two-tailed Student’s t-test.
(TIF)

S3 Fig CD80 and CD86 expression in GMMAC and GMDC subsets. (a) Gating strategy of mBMDCs to GMMAC (MHCII^low^CD11b^hi^CD115^hi^) and GMDCs (MHCII^hi^CD11b^int^CD115^low^). mBMDCs were not pretreated (b), and first pretreated with NSAIDs (c) celecoxib, (d) diclofenac, or (e) ibuprofen for 6 hr, followed by a 2.5 hr TLR stimulation with LPS, CpG or R848. After overnight rest in fresh medium, maturation marker expression was analyzed using flow cytometry (n = 4). Mean Fluorescent Intensity (MFI) ± SEM of CD80 and CD86 in CD11c+ GMMAC (b) and GMDC (b-e) population. Statistical significance was calculated using a one-way ANOVA with Dunnett’s multiple comparison test, medium versus rest.
(TIF)

S4 Fig TLR induced cytokine production by mBMDCs and moDCs. mBMDCs (a, n = 4) and moDCs (b-c, n = 5–6) were first pretreated with NSAIDs for 6 hr, followed by a 2.5 hr TLR stimulation with LPS or R848. After overnight rest in fresh medium (a-b) and directly after pulse (b-c), cytokines IL-6, IL-10, IL-12 (a-b), and TNF-α (a-c), were measured using ELISA. Results are shown as means with SEM. Statistical significance was calculated using Mixed-effects analysis with Dunnett’s multiple comparisons test, on raw data. (b) Detection limit for hIL-12 is 50–4000 pg/ml.
(TIF)
