# Supplementary figures and images for: NSAIDs affect dendritic cell cytokine production
Source: PLoS One. 2022 Oct 13;17(10):e0275906. doi: 10.1371/journal.pone.0275906 (PMC9560552; doi:10.1371/journal.pone.0275906)

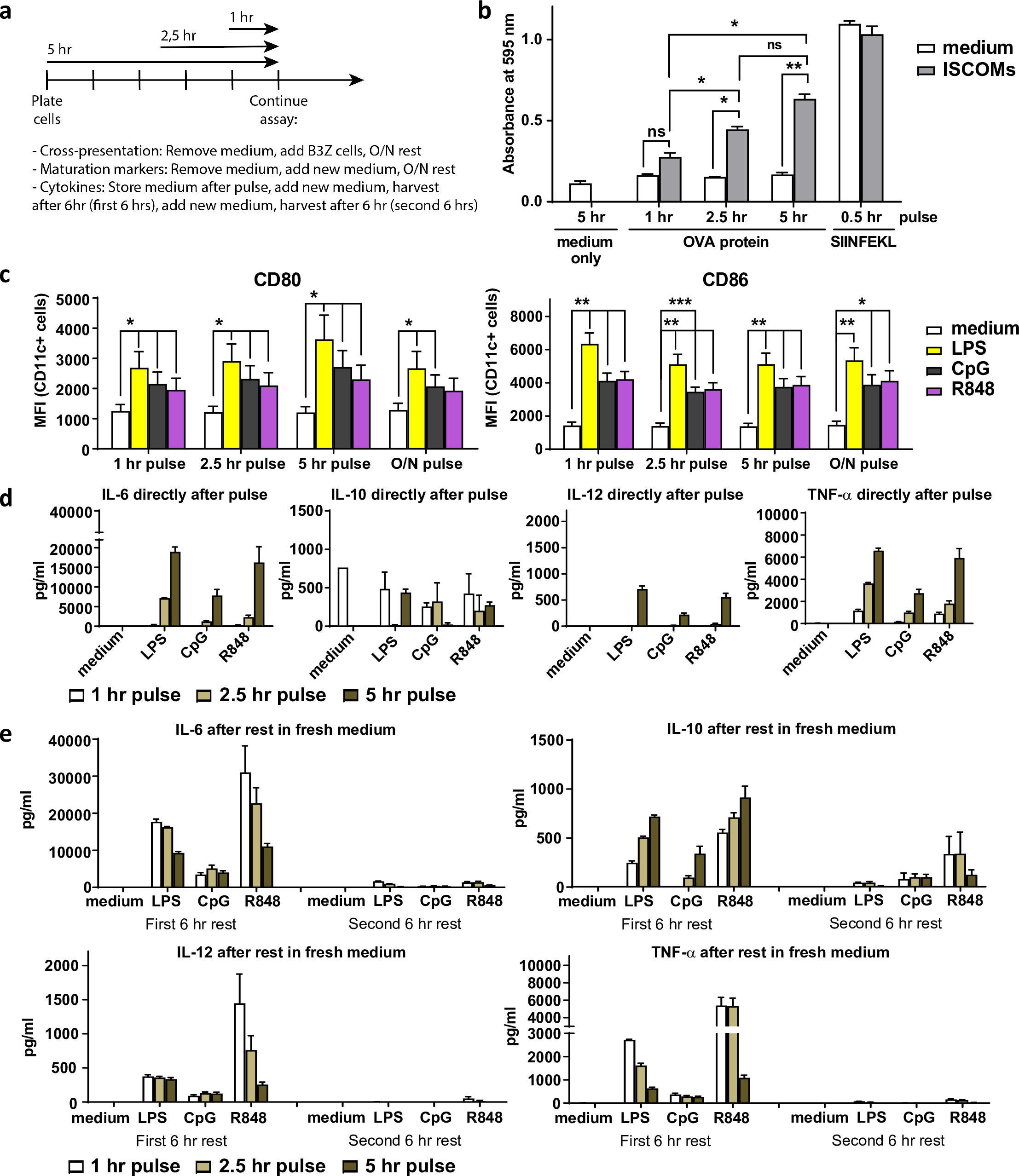

Supplement: S1 Fig — (a) Experimental design with different incubation durations and stimuli. (b) mBMDCs were treated with OVA protein and ISCOMs for indicated durations, and then co-cultured with B3Z T cells for 18 hr. As a positive control for viability and MHC-I levels, mBMDCs were pulsed with OVA peptide (SIINFEKL) 0.5 hr before coculture with B3Z T cells (n = 3). Statistical significance calculated using 2-way ANOVA, Sidak’s multiple comparisons test (for medium versus ISCOM), Tukey’s multiple comparisons test (for ISCOMs versus ISCOMs). mBMDCs were stimulated with LPS, CpG, or R848 for indicated durations (c-e). After overnight rest in fresh medium (for 1, 2.5, and 5 hr pulse) or overnight stimulation without rest in fresh medium (O/N stimulation), maturation marker expression in CD11c+ cells was analyzed using flow cytometry (n = 5). Statistical significance was calculated using a one-way ANOVA with Dunnett’s multiple comparison test, medium versus rest. Directly after the pulse (d), and during the first and second 6 hr after refreshment of the medium, cytokines IL-6, IL-10, IL-12 and TNF-α were measured using ELISA (n = 2–3). (b-d) Results are shown as means with SEM. (TIF) [file pone.0275906.s001.tif]

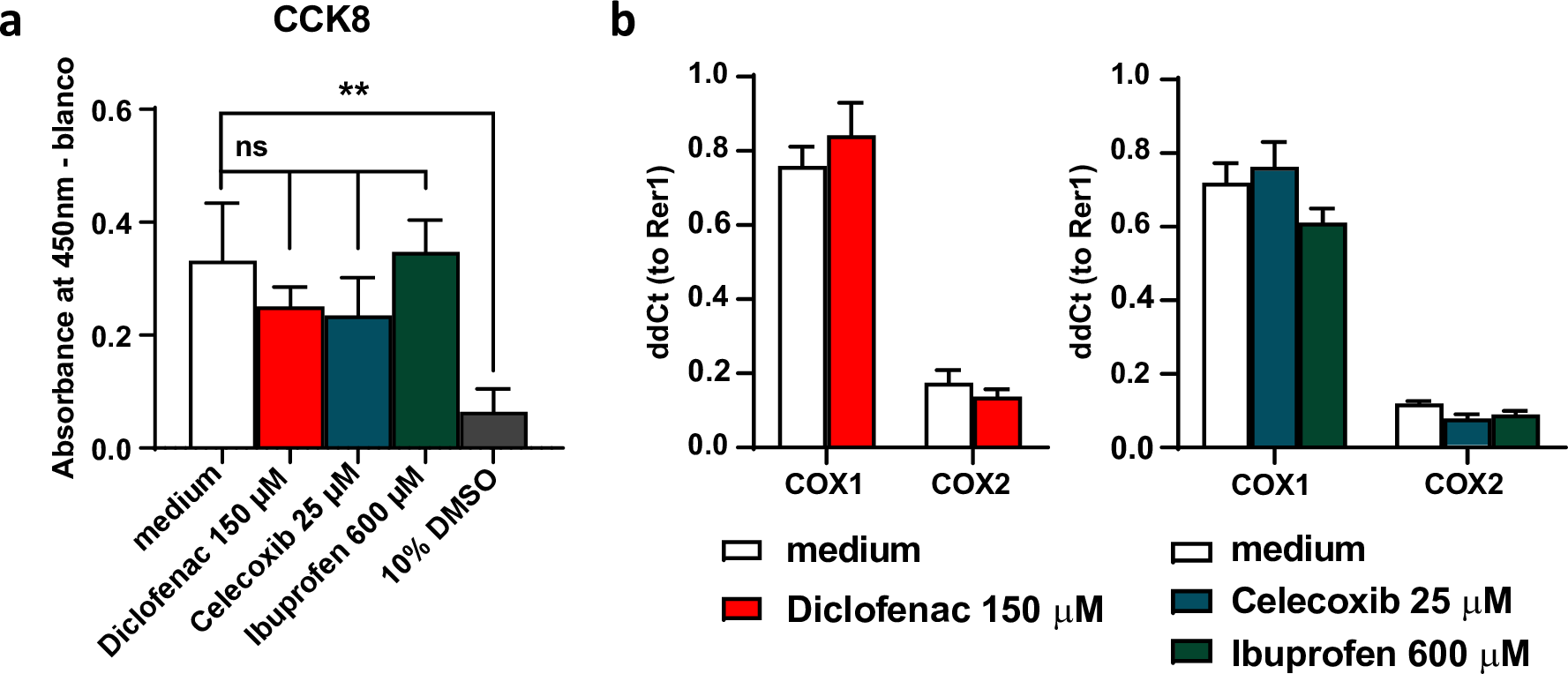

Supplement: S2 Fig — (a-b) mBMDCs were treated for 6 hr with NSAIDs. (a) CCK8 assay as a read out for cell viability and metabolic activity, after 6 hr stimulation with NSAIDs. Raw data–blanco depicted (n = 4). Statistical significance was calculated using one-way ANOVA with Dunnett’s multiple comparisons test, medium versus rest. (b) RT-QPCR was performed for mRNA expression of COX1 and COX2 (mBMDCs, n = 3–4). Results are shown as means with SEM. Statistical significance was calculated using a two-tailed Student’s t-test. (TIF) [file pone.0275906.s002.tif]

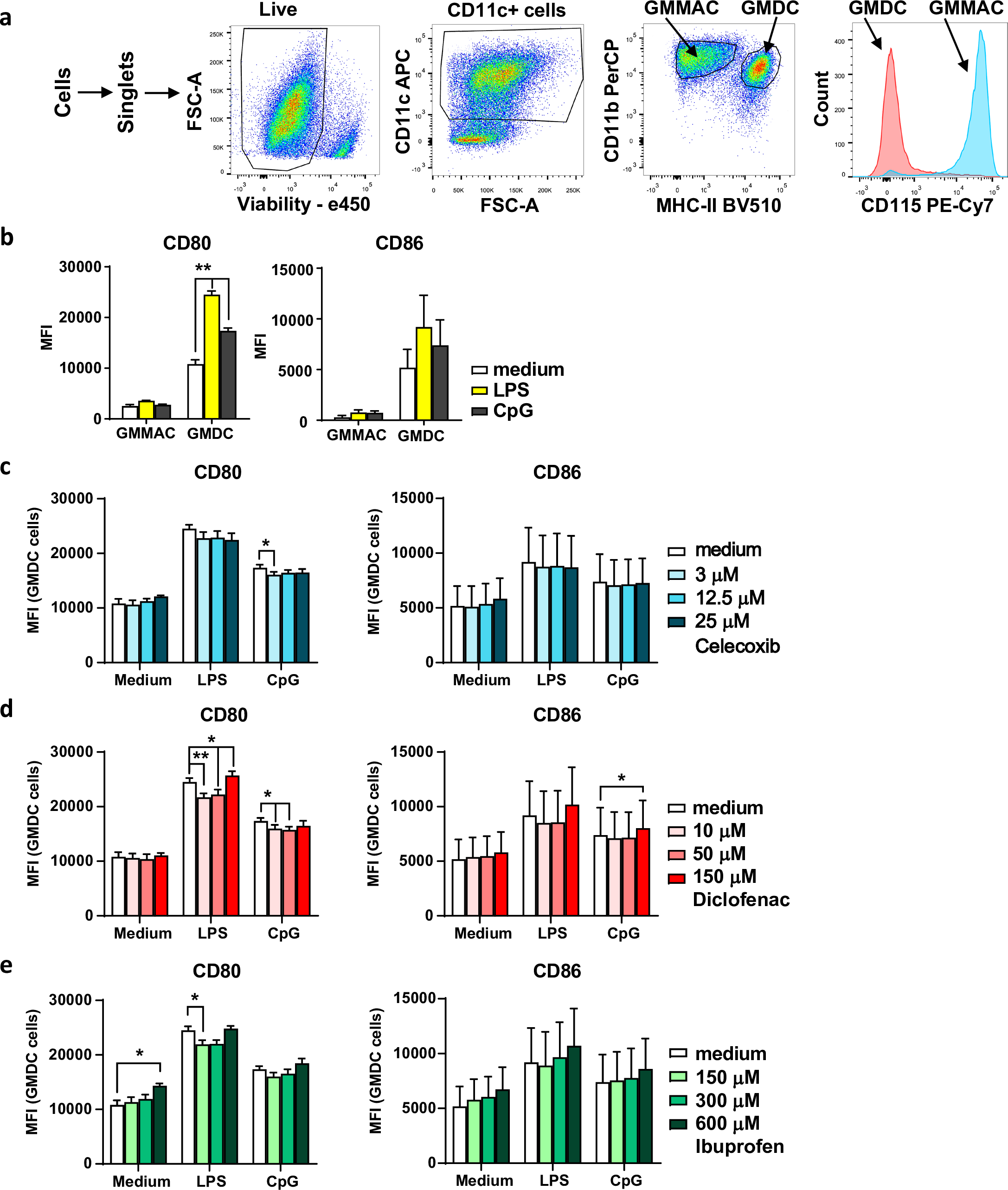

Supplement: S3 Fig — (a) Gating strategy of mBMDCs to GMMAC (MHCIIlowCD11bhiCD115hi) and GMDCs (MHCIIhiCD11bintCD115low). mBMDCs were not pretreated (b), and first pretreated with NSAIDs (c) celecoxib, (d) diclofenac, or (e) ibuprofen for 6 hr, followed by a 2.5 hr TLR stimulation with LPS, CpG or R848. After overnight rest in fresh medium, maturation marker expression was analyzed using flow cytometry (n = 4). Mean Fluorescent Intensity (MFI) ± SEM of CD80 and CD86 in CD11c+ GMMAC (b) and GMDC (b-e) population. Statistical significance was calculated using a one-way ANOVA with Dunnett’s multiple comparison test, medium versus rest. (TIF) [file pone.0275906.s003.tif]

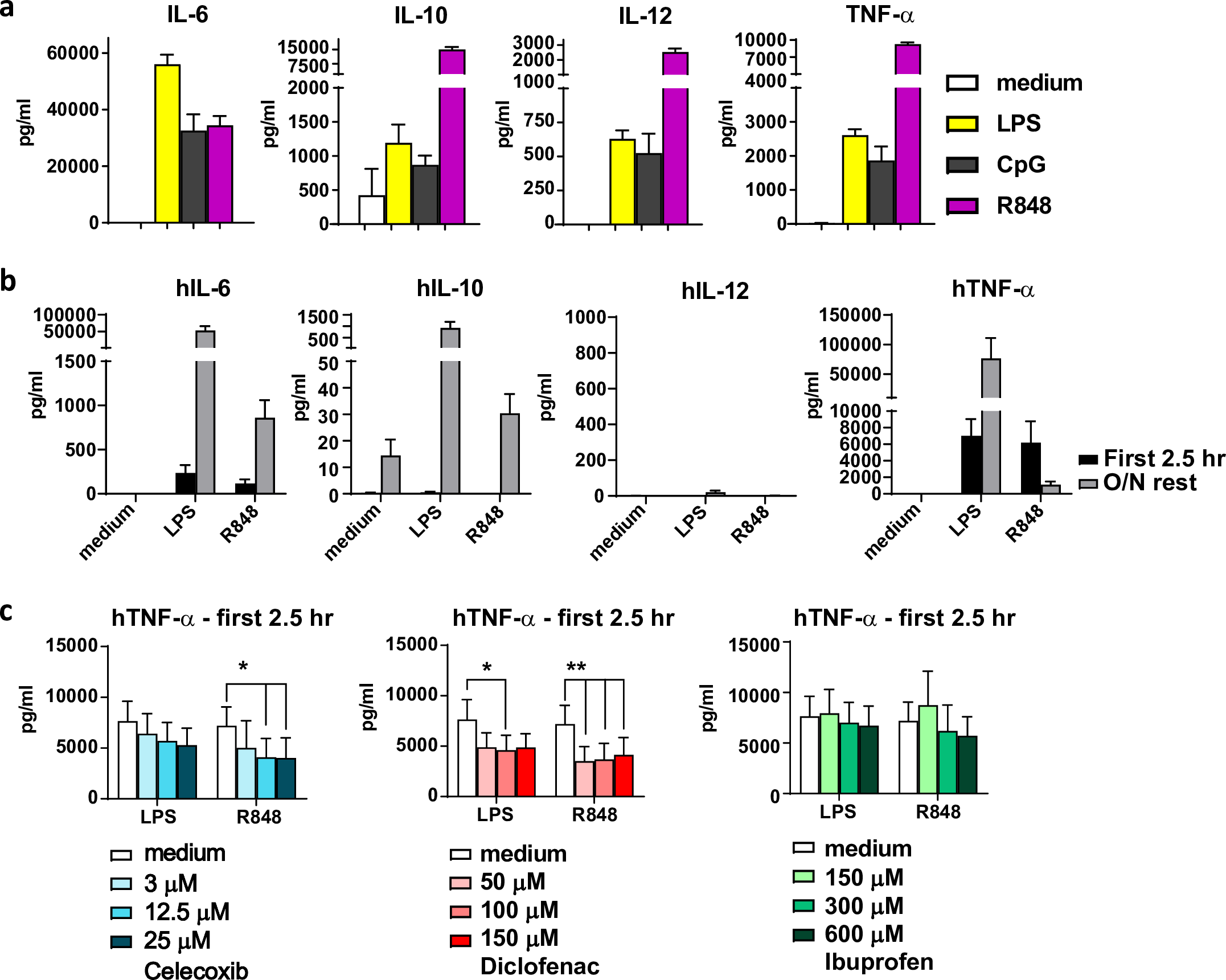

Supplement: S4 Fig — mBMDCs (a, n = 4) and moDCs (b-c, n = 5–6) were first pretreated with NSAIDs for 6 hr, followed by a 2.5 hr TLR stimulation with LPS or R848. After overnight rest in fresh medium (a-b) and directly after pulse (b-c), cytokines IL-6, IL-10, IL-12 (a-b), and TNF-α (a-c), were measured using ELISA. Results are shown as means with SEM. Statistical significance was calculated using Mixed-effects analysis with Dunnett’s multiple comparisons test, on raw data. (b) Detection limit for hIL-12 is 50–4000 pg/ml. (TIF) [file pone.0275906.s004.tif]
